# Enabling Heart Self-Monitoring for All and for AAL—Portable Device within a Complete Telemedicine System

**DOI:** 10.3390/s19183969

**Published:** 2019-09-14

**Authors:** Andrés-Lorenzo Bleda, Francisco-Manuel Melgarejo-Meseguer, Francisco-Javier Gimeno-Blanes, Arcadi García-Alberola, José Luis Rojo-Álvarez, Javier Corral, Ricardo Ruiz, Rafael Maestre-Ferriz

**Affiliations:** 1 CETEM-Technologic Centre of Furniture and Wood of Region de Murcia, 30510 Yecla, Spain; r.maestre@cetem.es; 2 Departament of Internal Medicine, Murcia University, 30001 Murcia, Spain; francisco.melgarejo@goumh.umh.es (F.-M.M.-M.);; 3 Arrhythmia Unit, University Hospital Virgen de la Arrixaca, 30120 El Palmar, Spain; 4 Murcian Institute of Biosanitary Research Virgen de la Arrixaca, 30120 El Palmar, Spain; 5 Departament of Communication Engineering, Miguel Hernández University, 03202 Elche, Spain; 6 Department of Signal Theory and Communications and Telematic Systemss and Computation, Rey Juan Carlos University, 28943 Fuenlabrada, Spain; joseluis.rojo@urjc.es; 7 Center for Computational Simulation, Universidad Politécnica de Madrid, 28223 Pozuelo de Alarcón, Spain; 8 RGB Medical Devices, 28037 Madrid, Spain; jcorral@rgb-medical.com (J.C.); rruiznolasco@rgb-medical.com (R.R.)

**Keywords:** ECG, arterial blood pressure, sensors, e-health, portability, atrial fibrillation detector, QRS detector

## Abstract

During the last decades there has been a rapidly growing elderly population and the number of patients with chronic heart-related diseases has exploded. Many of them (such as those with congestive heart failure or some types of arrhythmias) require close medical supervision, thus imposing a big burden on healthcare costs in most western economies. Specifically, continuous or frequent Arterial Blood Pressure (ABP) and electrocardiogram (ECG) monitoring are important tools in the follow-up of many of these patients. In this work, we present a novel remote non-ambulatory and clinically validated heart self-monitoring system, which allows ABP and ECG monitoring to effectively identify clinically relevant arrhythmias. The system integrates digital transmission of the ECG and tensiometer measurements, within a patient-comfortable support, easy to recharge and with a multi-function software, all of them aiming to adapt for elderly people. The main novelty is that both physiological variables (ABP and ECG) are simultaneously measured in an ambulatory environment, which to our best knowledge is not readily available in the clinical market. Different processing techniques were implemented to analyze the heart rhythm, including pause detection, rhythm alterations and atrial fibrillation, hence allowing early detection of these diseases. Our results achieved clinical quality both for in-lab hardware testing and for ambulatory scenario validations. The proposed active assisted living (AAL) Sensor-based system is an end-to-end multidisciplinary system, fully connected to a platform and tested by the clinical team from beginning to end.

## 1. Introduction

A clear trend of population aging has been emerging in developed nations during the last decades and it is recently accelerating. This scenario poses a challenge that will impact our societies forever bringing profound changes, especially related to economic sustainability due to the enormous resources required to provide the necessary care for older adults. The higher growth of the 65-and-over population segment, together with the decreasing birth rate, is inverting the population pyramid in most developed economies and it is becoming a challenge to build an adequate care system to face these new dynamics. According to the World Health Organization (WHO), this trend has been identified as one of the biggest challenges for our society [1], which needs to be addressed so that the necessary services can be provided at manageable cost [2]. Policy makers have realized this situation and they are doing a great effort to support initiatives and create new services for the older population, often based on technological solutions that promote their independence and provide new intelligent functionality for helping them on their daily living needs and health issues. The elderly is the most prone population group to suffer cardiovascular diseases (CVDs) such as heart attacks, strokes and arrhythmias. Up to 17.9 million people die each year from CVDs (31% of deaths worldwide) and more than 75% of these deaths are due to heart attacks and strokes [3]. Frequent cardiac monitoring at home can help to identify CVDs and prevent future cardiovascular problems and even risk of death can be predicted with the electrocardiogram (ECG) [4]. To that end, telemedicine devices operated by patients themselves from their own homes are a great solution, since they do not require continuous medical professional assistance. Traditional medical-grade ECG is obtained by attaching electrodes (between 3 and 10 of them) on specific points of the body skin around the chest [5,6] and it is typically carried out and analyzed by medical personnel with the necessary knowledge and experience. However, this type of ECG can be sometime cumbersome and unpractical for older people to use. Recent technology developments have created social environments where we can find a number of commercially available devices for ECG recording or also for ABP signal measurement and all of them for home usage. However, and to our best knowledge, none of the existing non-ambulatory devices is able to record, measure, and process index arrhythmia diagnosis and to transmit that information to a centralized heath service and, even more, we seldom find a complete system that has been clinically validated.

In this paper, we present the VitalMob telemedicine system for heart monitoring, which was developed as part of the VitalMob Multidisciplinary Research Project (see Funding right after Section 6) and we describe the portable device designed and built, as well as its companion smartphone app. The device is capable of recording high quality ECG signals and measuring arterial blood pressure (ABP). The so-called VM1 device is controlled via a Bluetooth-LE connection by said smartphone app, which also guides the user through the multiple steps of the measuring process. Device and app were both jointly designed for maximum ease of use, so that most of common older person could use it with no external aid. If assistance were necessary, any relative or friend would be able to help with no previous training. The ECG signal can be visualized during the measuring in real time by its user, who can decide whether to upload to the cloud after recording it or to repeat the process if something went wrong. Similarly, the ABP measurement is also shown and it can be repeated before its sending. Once in the cloud, data are analyzed by specifically developed algorithms that look for potential problems and anomalies (e.g, arrhythmias). If anything unusual is detected, the doctor is warned so that she/he can inspect the recorded data through any internet-capable device and visually confirm if it is a real problem and decide how to proceed.

The present work is the result of a multidisciplinary research project and the complete system has a part of integration of communication and transmission of the ECG and a tensiometer measurements, within a patient-comfortable support, easy to recharge and with a multi-function software, all of them adapted for elderly people. The novel contributions of the present work can be summarized as follows:

- First, the two variables (ABP and ECG) are simultaneously measured in an ambulatory environment, which to our best knowledge is not available in the clinical market.
- Second, the proposed system is an end-to-end one, fully connected to a platform and is tested and validated by the clinical team from beginning to end.
- Finally, the signal processing algorithms are created and validated in this clinical integrated scenario, rather than in in-lab or in-silico prototypes, which yields relevant information about the scope of the designed algorithms in operating mode.

The paper is organized as follows. First, in Section 2, the state of the art is presented and discussed in relation to VM1 device and our proposed algorithms. Then, an overview of the materials and methods used in this work is described, including the VM1 device, its smartphone app and the remote e-health platform. Section 3 presents a brief introduction to some of the algorithms that can be found in the literature and they cover the topic of wearable devices signal quality, QRS-detection in wearable scenarios and multimodal derivation of heart rate signal. Section 4 presents and analyzes the experiments and results that were used for testing the VM1 device and the ECG analysis algorithms. Finally, Section 5 is devoted to the presentation of the main conclusions of this work and how it could evolve in the future, followed by a mention of the patent that has been derived from this work.

## 2. State of Art

In this section, we include a brief review of some relevant background works related to the ECG processing, an introduction to the commercial telemedicine monitoring devices and we finalize by presenting an almost existing consensus on the process followed in ECG processing existing work.

### 2.1. Literature on ECG Processing

Starting with the background on ECG processing, we incorporate next a subset of reference articles covering several key topics in this subject, namely, signal quality in wearable devices, QRS-complex detection in high-noise scenarios and algorithms for multimodal heart rate estimation.

Despite the extensive and even growing literature related to ECG processing, an illustrative, although not extensive selection of works, needs to be highlighted for an integrated and comprehensive view of the subject. We initiate this review addressing several representative papers that have analyzed the required signal quality for later enhanced processing. Example of this is presented in [7], where the authors proposed to classify the ECG noise condition into five levels according to medical criteria on the clinical severity of the noise. Expert clinicians performed this classification manually. First, a specifically designed software represented the ECG. Then, the experts selected groups of three beats for further classification. Finally, the experts classified segment according to several rules, namely, noise free when segments were considered without noise, low noise when segments exhibit noise but waves were visually isolated, moderate noise when just QRS complexes were the only waves visually detected and hard noise when waves were unrecognizable. In this work, the authors presented a tailored filter to remove the so-called large amplitude noise that makes the signal unreadable due to signal loss or amplifier saturation. In [8], the authors present a number of different variables to reflect the recorded signal quality. These measurements can be divided in three groups. First, the lead-fall detection, where they detect if the ECG signal exists. Second, the signal quality indices, which are a number of different measurements that consider as ECG signal features the number of QRS complexes detected by two detector algorithms. Third, the morphology of consecutive QRS, as well as the difference between consecutive RR intervals. Finally, they also considered certain non-linear indexes, such as sample entropy, fuzzy measure of entropy or Lempel-Ziv complexity, among others. According to these values they generate a five-level signal-quality scale and they trained a Support Vector Machine classifier, reaching up to 97.9 (96.4) % accuracy in readable (non-readable) signal segment classification.

Entering into the existing publications related to wearable devices, an interesting contribution to this field can be checked in [9], where the authors tested one of these devices for ECG monitoring manufactured by OMgarments^TM^ and explained the signal quality classification system implemented by said company. The proposed method splits the total registers in 15-minute strips and each strip is processed to extract the noise level and the heart rhythm. After that, the last 7 s of each strip are also extracted to compute a morphology analysis of P waves, QRS complexes and T waves. Using these previously computed measurements, the algorithm classifies the strips into three levels according to their signal quality, which are the following ones: Dominant, where the where the rhythm can be extracted from 75–100% of all beats from the strip and the P, QRS and T waves can be delineated; Significant, where the rhythm can be extracted from 50–75% of all beats from the strip; And inadequate, where the rhythm ca be extracted from less than 50% of all beats from the strip.

The multimodal analysis, understood as the systems including at least ABP and ECG signals, has gained interest in the recent years and it is often now incorporated in many commercial devices. This is especially true in the case of wearables. In these cases, as the recordings are not performed in an ambulatory or controlled environment, noise components should be specially considered. In [10], a robust heart beat detector for multimodal records is presented. The ECG signal is used jointly with pulsatile signals, such as blood pressure and pulmonary artery pressure. An initial separate multi-step detection processing is proposed for each registered signal, followed by a second later regularity test where the detections are compared. In the proposed algorithm, in case of irregularities are identified, detection is based only on pulsatile detections, whereas ECG and pulsatile detections are merged otherwise. Another interesting contribution to this field is presented in [11], where a simple algorithm for heart beat detection based on multimodal analysis was evaluated. This algorithm can be divided in three main parts, namely, ECG and ABP pulse detection, computation of signal quality measures and creation of a beat array. In the first step, several QRS-complex detectors were tested, namely, *gqrs, coqrs, eplimited, jqrs,* and wavelet. The *wabp* algorithm was used to detect the peaks in the ABP signals and similar strategies were used for stroke volume and for the photoplethysmogram. Then, some signal quality metrics are computued based on a trade-off between two different QRS-complex detectors. Finally, the heart rate signal is created by selecting the correct heartbeat according to the value of signal quality metrics. In [12], the authors present a three-stage robust algorithm to derive the heart rate signal by using different biological measured signals, namely, the ECG, the ABP, the electroencephalogram, the electro-oculogram and the electromyogram. In the first step of this algorithm, the R-peaks and the pulses are detected by using *gqrs, wabp,* and *SSF & TKE* methods, according to the source of the signal. In the second step, a signal quality index is computed for each detected beat and it is calculated as a product of a heart-rhythm factor, which takes into account the deviation and the mean of a series of neighbor beats and a beat-deviation factor, which takes into account the deviation among the beat and its neighbors. Finally, a voting method is implemented to select the correct beats among the detected ones.

As mentioned earlier, noise in wearable devices is a relevant issue and it has ben addressed differently from different authors. However, after thousands of papers issued showing different QRS detection methods, this remains an open issue or at least a topic that comes to be something that could be always improved, even in a controlled ambulatory environment. This detection is almost more challenging when new devices are used and environments are open to additional effects. Different research groups have faced this catch and hereafter we summarize some representative works issued in this field. In [8], the authors tested ten of the most used QRS-complexes detection algorithms, namely, Pan-Tompkins, Hamilton mean, Hamilton median, RS slope, sixth power, finite state machine, U3 transform, difference operation, *jqrs* and optimized-knowledge based. These algorithms were benchmarked over four representative databases, including high-and-poor signal quality ECG database from 2014 Physionet challenge, normal sinus rhythm and arrhythmia ECG database from MIT-BIH, pacemaker rhythm ECG database from MIT-BIH and telehealth ECG database from TELE database. Results showed that optimized knowledge-based was the best algorithm for telehealth record processing, with 80.43% in F1 score. In [13], a QRS-complex detector is proposed based on a tailored version of the Pan-Tompkins algorithm, which includes multi-lead processing to enhance its performance. The authors tested this new algorithm over several public and private ECG databases, namely, private vPredict database compounded by 24-h records from heart failure patients, public arrhythmia database from MIT-BIH, public noise stress database from MIT-BIH, public long-term ST database from MIT-BIH, public long-term atrial fibrillation (AF) database from MIT-BIH, public long-term database from MIT-BIH, public INCART database from St. Petersburg Institute of Cardiological Techniques and private PREMARIS database compounded by 7-day records. This algorithm exhibited accuracy above 96% in each used database, though in the noise stress database worst cases the algorithm reached accuracy above 80%. In [14], a novel method was proposed to detect the heart rate by using three unobtrusive ECG signals and three unobtrusive optical pulse signals, also combining them by means of robust Bayesian fusion. The ECG signals were recorded by using capacitive sensors and the optical sensors were based on photoplethysmography. The QRS-complexes were detected using the Hamilton open-source algorithm and the optical signal pulses were detected by using a maximum-value detector based on the Savitzky-Golay filter. The authors presented three different fusion algorithms, namely, median of all sensors, selection of the best sensor according to a quality index and Bayesian fusion, which is based on Bayes Theorem. Their results showed that the use of Bayesian fusion achieves an error lower than 2 beats per minute along the 80-90% of total test duration.

### 2.2. Telemedicine Monitoring Devices

The Vitalmob system scope is the acquisition of two highly valuable medical parameters related to heart health measurements, namely, ECG and ABP. The two main groups of ABP measurement techniques are grouped into invasive and non-invasive [15]. Invasive techniques have the major drawback of requiring medical intervention to place a cannula needle in an artery and close medical supervision and they are much more accurate than non-invasive ones, thus they are mostly used for hospitalized and critically ill patients. On the other hand, non-invasive techniques are quicker and simpler than invasive ones, they do not require any expertise and they have no difficulties for end users who are even able to make the measurements personally with portable devices. Automatic ABP monitors are very common and well-known nowadays, even for personal use at any age. Many different models are cheaply and widely available on the market [16,17]. These devices are usually very easy to operate for users, sometimes they allow data to be stored digitally and even they can be connected with a mobile app for an interface [18,19]. The VM1 integrates a cuff-based ABP measurement system as part of its functionality. The cuff is connected to the main device chassis and it can be used in a straightforward and safe way.

On the other hand, ECG is crucial for monitoring a person’s heart and vascular health. A standard 12-lead ECG is routinely used for clinical monitoring [20] but this is impractical for use cases where portability and simplicity are important. The first portable ECG monitors were mainly for medical ambulatory use and handled by professionals and for that reason, this type of devices often are more complex to operate but during recent years, information and communications technology (ICT) advances are bringing this technology closer to end users. Nowadays, two-electrode ECG monitoring is widely accepted for ECG portable devices [21]. There are many different commercial examples, such as the AliveCor system [22], which consists of a pocket device for ECG measurements capable of obtaining normal heart rhythm and detect AF. Similarly, Chekme Lite device from Viatom [23] is a portable ECG recorder with pulse-oxymeter. Other examples are the SnapECG handheld-ECG recorder [24] or the QardioCore wearable ECG monitor [25]. Most of them are stand-alone devices of Holter type and they are not capable of sending the acquired data to any external server in real time. One important different among others, of the device presented in this work, the VM1, is the fact that this device could work with up to four electrodes, providing additional features and functionality, from other mentioned with two and one single operational mode. Detailed information in that regard is included in next section.

### 2.3. Processing Algorithms

According to existing literature, ECG analysis algorithms can be divided in three main parts: First, in the *signal accommodation* stage, the signal is processed in order to remove or at least to reduce the total amount of present noise; Second, in the *beat detection* stage, the beats are detected in order to extract the heart rate signal, which is inversely proportional to the time among peaks; Finally, in the *postprocessing* stage, different ECG features are deeply analyzed in order to detect alterations that can indicate the presence of some diseases. In this work, we focused on rhythm alterations scoped by heart rate variability (HRV) analysis techniques working on the stored ECG. Note that this storage allows the inclusion of further digital processing, such as morphological analysis techniques, though they were not considered within the scope of the current end-to-end VM1 system.. We next summarize the most convenient options available in the literature and the following sections specify the chosen options and their parameters in our system.

As part of the first phase, the noise present in ECG is often classified in several main groups, according to its nature and origin, namely, baseline drift, powerline interference, muscular noise (electromiogram or EMG) or body movement [26]. Several or even all of these contributions can be present simultaneously in a recording. The baseline drift, which appears as a low-frequency changing-bandwidth signal that modulates the ECG amplitude, is generated by the effects of electrode-contact impedance, the movement associated to patient respiration and patient body movements. Its frequency is typically lower than 0.5 Hz and the main techniques to reduce it are the following: Linear time-invariant filters, which consist of high-pass filters with 0.5 Hz cut-off frequency; Linear variant filtering, which consists of high-pass filters with a cut-off frequency that is inversely proportional to the instantaneous RR interval; And polynomial fitting, which consists of approximating the drift with a smooth curve, e.g, using a cubic spline interpolation and then substracting it.

The powerline interference, which appears as sinusoidal-like fluctuations at powerline frequency in each country and its harmonics, is created by problems in the recorders, grounding or shielding. This kind of noise, which inserts in the frequency band of interest for the ECG, difficults the low-amplitude waveform interpretation, such as P or Q waves. The main used techniques to reduce it are the following: Linear time-invariant filtering, which consist of a notch filtering centred in powerline frequency; Non-linear time-invariant filtering, which consists in substracting from the signal a sinusoid generated with a non-linear filter that updates its parameters according to an error function; And adaptative filtering, which consists of estimating the powerline noise present in the ECG and subtracting it.

The EMG noise, which appears as peaks up to 10% the ECG amplitude, can be specially intense in Holter-recorded ECG and its bandwidth goes from 20 to 2 KHz so that it strongly overlaps the ECG signal. Therefore, it constitutes a relevant challenge in this kind of signal denoising. Standard filtering techniques could be applied, with caution of avoiding negative shaping distortion of the actual QRS and also statistical filtering by averaging QRS complexes can be used in some applications, with the only requirement of having multiple properly correlated beats.

For the beat detection stage, we can divide the usual algorithms in several main groups, according to the used method for beat position extraction, namely: Digital filtering methods, which are based on applying different kinds of filters to isolate the main peak of the beat (which is the R-wave) and use a threshold to detect its position [27]; Wavelet transform methods, which are based on detecting the peaks by calculating the degree of uniqueness in different scales of said transform [28]; Split spectrum methods, which split the signal in different frequency band and perform a recombination to create a new one where the beats are enhanced in order to detect them by thresholding [29]; and statistical machine learning methods, which are based on error function maximization predicting the base-line, in other words, they detect the peaks as the point with larger error values [30].

Nowadays, existing HRV analysis techniques can be divided into three main groups [31], namely, time domain methods, which are compounded by statistical and geometrical techniques; frequency domain methods, which are divided in short-term and long-term analysis methods; finally, non-linear methods, which are compounded by several types of measurements based on chaotic or fractal signal models, such as random walk, approximate entropy or bispectrum, among others. In this work, a number of time domain methods are used and the most relevant ones are next explained. The time domain methods based on statistical techniques perform an statistical analysis of the temporal difference between each detected beat, which is called RR or NN interval series according to excluding or not the non-sinusal beats. Some eamples of basic indices in this group are the average heart rate, the difference between the longest and shortest interval, the difference between night and day heart rate and the NN interval standard deviation. On the other hand, among the geometrical methods we can highlight the following ones: The triangular index, which measure the triangularity of the RR interval histogram; The Lorenz-Poincaré diagram, which is a point cloud representation by each RR interval based on its precedent; And the logarithmic coefficient, which represents the coefficient from the negative exponential curve which is the best approximation of the histogram of absolute differences between adjacent NN intervals, among others.

## 3. Material and Methods

In order to allow a better comprehension of this work, this section is structured as follows. First, a whole system overview is performed. Second, the different hardware parts of the novel device are devoted, focusing on its more relevant features. Third, the companion smartphone application, which masters the VM1 operation, is presented and its behaviour is explained. Finally, the developed remote e-health platform is devoted, focusing on each implemented ECG processing and analysis algorithms.

It should be mentioned that, from a functionality standpoint, this device could be used in many different situations, although the operational basic design was twofold: First, and especially devoted to the elderly, for frequent registry in domestic environments allowing the in-home semi-supervised high quality bio-signal registry; And second, an on-demand event-Holter functioning-mode is also possible, where the user can be instructed by clinicians and health staff to initiate manual registry in case of any identified health event appearing. Therefore, in the case of the patient using the device under clinical advice and supervision, it would be up to medical doctors to orientate the purpose or to suggest the required posology and indications, setting the time of the day, the frequency or the physical feeling events that the patient should be aware of, in order to initiate a manual recording.

**Figure 1:**
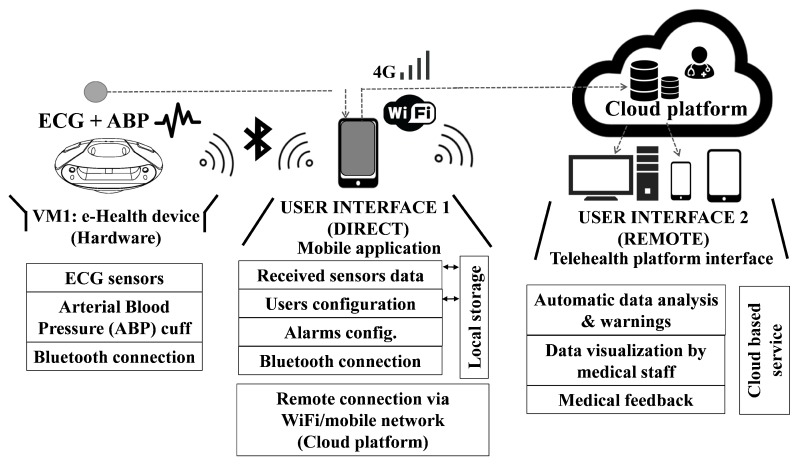
System architecture. e-Health device (VM1) functionality and two available user interfaces. (Interface 1) Mobile application directly connected to VM1 via Bluetooth. (Interface 2) Telehealth cloud based platform and services.

For the end user, the VM1 portable monitor is the core of the VitalMob system, which has been designed with a very ergonomic round and wide shape that includes two separate areas for placing the hands over the two standard functioning ECG electrodes. The VM1 is a sensor device capable of ECG monitoring and it includes an ABP cuff connected via Bluetooth^TM^ to a mobile application that provides the user interface, carries out the necessary processing and storing and also displays the acquired data in real time during the measuring process. The application also acts as a gateway through which the acquired data can be sent to a remote cloud platform for automatic medical analysis. An overview of whole e-health system can be seen on Figure 1.

### 3.1. VM1 E-Health Device

**Figure 2:**
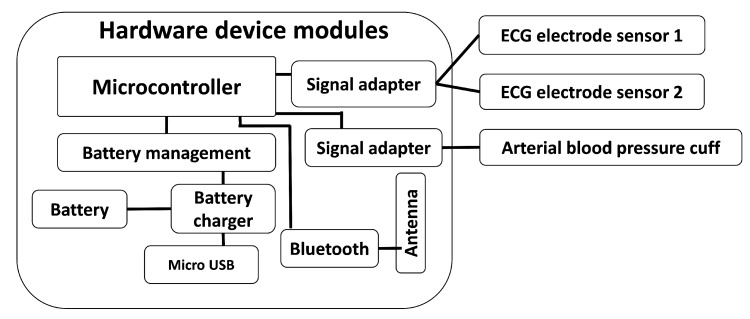
Hardware modules. Main module (microcontroller, battery management, Bluetooth communication and signal adapters) and external sensor modules (ECG and ABP).

Many different requirements were identified during the research and the development activities of the VM1. Most of them were related to the hardware design of the system (not only the electronics but also the design of the external physical case). Specifically, the VM1 includes a portable ABP cuff with a compressor and a two-electrode ECG measuring device, which can be extended up to four electrodes by means of two additional wired electrodes that can be plugged in the available connectors. It also includes other required hardware elements as seen in Figure 2), such as a battery, a wireless connectivity module (Bluetooth^TM^), a processor that orchestrates the rest of the system elements and the communication with the mobile application. This app stores the data of the measurements collected and sent by the VM1 and it controls the VM1 itself through the Bluetooth^TM^ communication interface.

**Figure 3:**
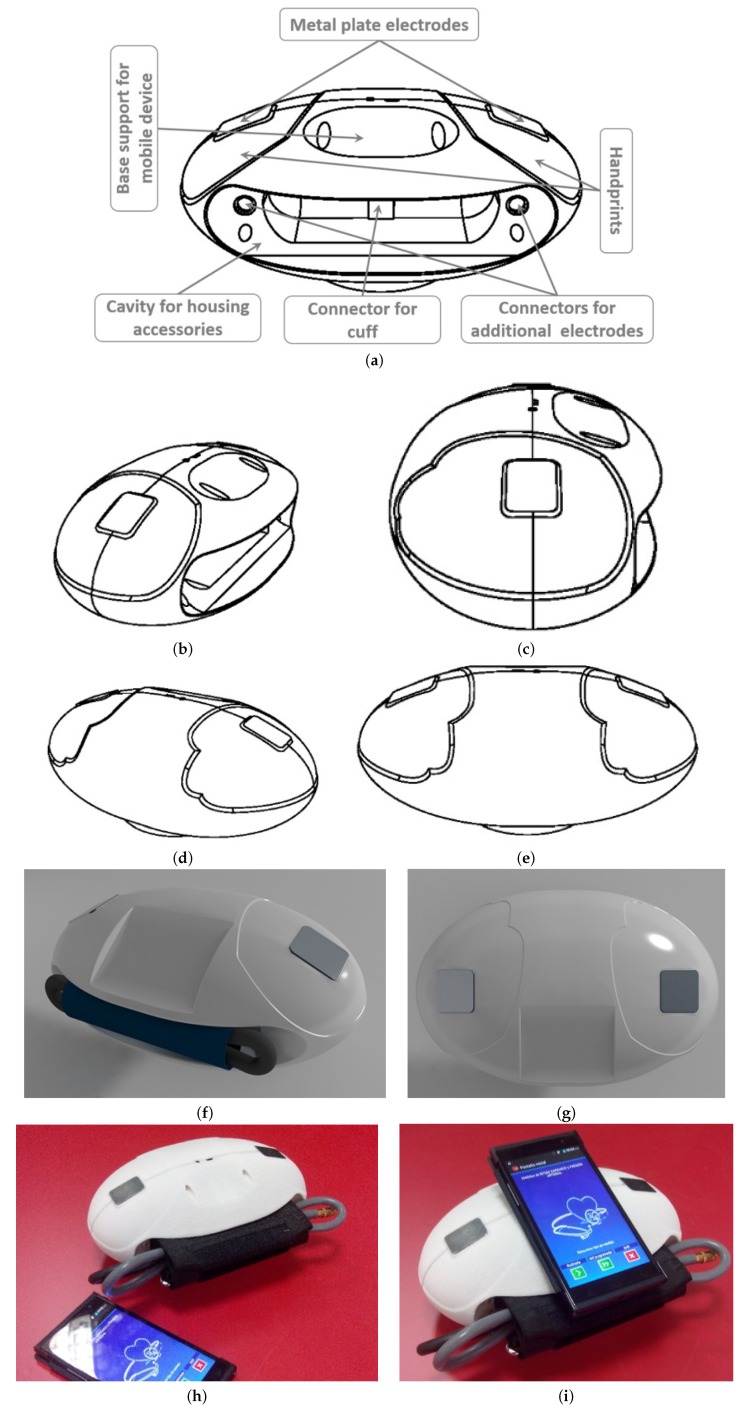
Different stages in the VM1 design from concept to prototype. (**a**–**e**) Portable and ergonomic design concept description and different views. (**f**,**g**) Case designed using standard CAD design software tools, such as Rhinoceros^®^ or 3D Studio Max^®^. (**h**,**i**) Final VM1 prototype made using precise state-of-the-art 3D printers.

An exhaustive, precise and innovative design has been achieved. The designed device has an oval shape and two metal plates electrodes are placed in the upper lateral areas of the device for ECG measurements. The electrodes are surrounded by handprints that indicate how to place the hands on top of the electrodes in order to obtain ECG measurements at the same time. The oval shape and handprints facilitate hands placement and reduce error possibilities during the measurement procedure, because hands position is unique, unlike other devices that require the placement of the electrodes in different parts of the body. The oval shape also facilitates ergonomics and comfort for users. The device can be placed on the user’s lap for reducing the risk of falling and breaking and it can also be hold and moved with one hand. The design stages from concept to prototype are shown in Figure 3. The VM1 has a flat wide base at its bottom to provide stability directly on almost any surface (for instance a table, a bed or the user’s lap). A holding base on which to place the mobile device is situated in the centre upper part of the portable device, facilitating the visualization and operation of the whole process during the acquisition of ECG and ABP measurements through the application. Nevertheless, the mobile device can be placed in another nearby location (such as a table) or even be controlled by another person at a distance, as long as the device and the mobile device are within the range of the Bluetooth^TM^ connection.

The VM1 design includes a large cavity in the middle part of the structure, under the support of the mobile device. This cavity has been designed to house measuring accessories, including additional connectors to two optional wired electrodes and their cables for contact in any part of the body, as well as the cuff for ABP. These optional wired electrodes can be connected to the VM1 through two additional connectors, as explained before. VM1 is versatile device enough to provide three modes of operation, namely, two embedded electrodes, two wired electrodes and two-by-two. In the first mode, they can be electrically connected to the metal electrodes located in the device upper zone. This is especially devoted for example in case the user needs an ECG but instead of going through a more uncomfortable process of directly placing the electrodes on her/his chest, the user just needed to locate her/his hands over the device. In the second mode, the previously mentioned additional wired electrodes can be used solely and properly placed it could be registered an almost a standard ECG—Lead I register. In addition, in the third mode, both previous modes can be combined giving up to a total of four simultaneously working electrodes. Thanks to these additional wired electrodes, in this third mode, the user can place them on the chest closer to the heart to collect signals that are more precise. These wired electrodes also provide better electrical skin contact to improve the ECG signal quality. It is important to notice that target users, the elder people, may suffer from hand tremors that in certain cases will provoke the signal not to be stable and to incorporate additional artifacts. In these cases, the double registry (2-by-2), when placing the wired electrodes at the same points that traditional chest ECG, would allow much better recording and denosing capabilities of the system. Specifically, the two additional connectors for electrodes are situated on the sides of the cavity, while the connector for the ABP cuff is located in the central part of the hole.

The VM1 aims to be used by people with heart monitoring necessities, especially elderly on their own without any additional assistance, hence, the device had to be ergonomic and user-friendly in home environments.

### 3.2. VitalMob App

**Figure 4:**
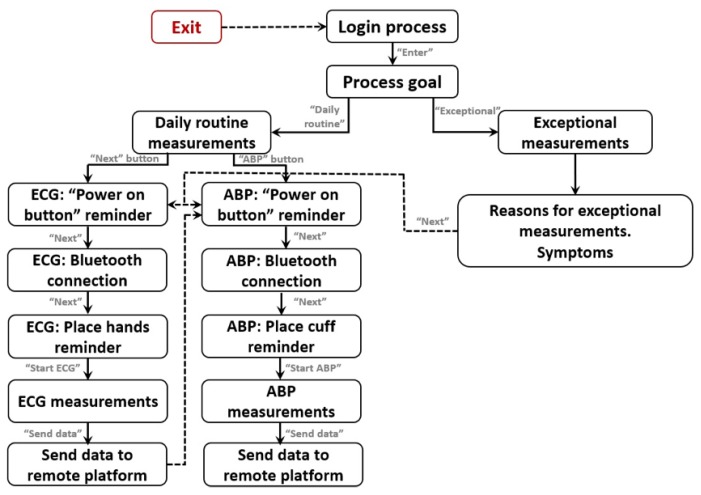
App flow block diagram.

The mobile app is a fundamental part of the VitalMob system allowing users to control the device, to store and visualize acquired data and to send it to a remote e-health platform. The main target end-users of the system are the elderly and it is necessary to take into account the limitations of this population sector. For example, they usually are not familiar with managing this type of applications and ICT devices. The app includes different functionalities, most of them are transparent to the user to facilitate its use and they are the following ones:

- *Login process* is the initial login screen. It is included to identify the user, so that user name and password have to be entered correctly. This is essential for a proper match between acquired data and user or patient identification. It should be noted that the system can be used by different users with a unique mobile phone and a login process prevents any potential error of data assignment to a wrong user.
- *Guided measurement procedure*. The app includes an easy and guided procedure for daily routine measurements with different screens until the ECG or ABP is acquired. This procedure is very simple and has easy-to-follow steps: (i) To remind the process goal (ECG or ABP) and to select daily or exceptional measurement; (ii) To Push power button of the VM1; (iii) To establish the Bluetooth^TM^ connection; (iv) To place the hands on the device or to put the cuff in the arm; (v) To wait to measurements finish; And (vi) to send data to platform. The complete app functionality block diagram and its control flow can be see on Figure 4.
- *Exceptional measurements*. The system can also be used when the user feels any pain or symptom (e.g., fatigue, vertigo, palpitations, chest pain, among others) In this case, the user introduces a description of his/her symptoms and this information is also sent to the remote e-health platform.
- *ECG measurements*. The user is able to visualize ECG data in real time during the measurement. This functionality is very useful for the user to decide whether to accept or reject the acquired ECG data. During the ECG measurement process the user can have any interruption and be forced to abandon the process and in this case, she/he can reject the acquired data and proceed with a new acquisition later.
- *ABP measurements*. The ABP measurement is a unique process that requires the proper placement of the cuff on the user’s arm while being in a comfortable posture. In case of any interruption or problem during ABP measurements, the user can reject the acquired data.
- *Data storage*. The app is configured to store the data in mobile memory in a file with *csv* extension. Sometimes the user does not want to send the data to the e-health platform immediately. In this case, data can be stored in the mobile phone to be sent later, for example, when a WiFi network is available.
- *Send data functionality*. The acquired data has to be sent to an e-health platform for remote automated analysis. The data can be sent using existing WiFi or data network connection from the mobile phone to the remote e-health server.
- *Cancel and exit*. The user can exit from the application at any point of the measurement procedure. All screens include an *exit* or *cancel* button.

**Figure 5:**
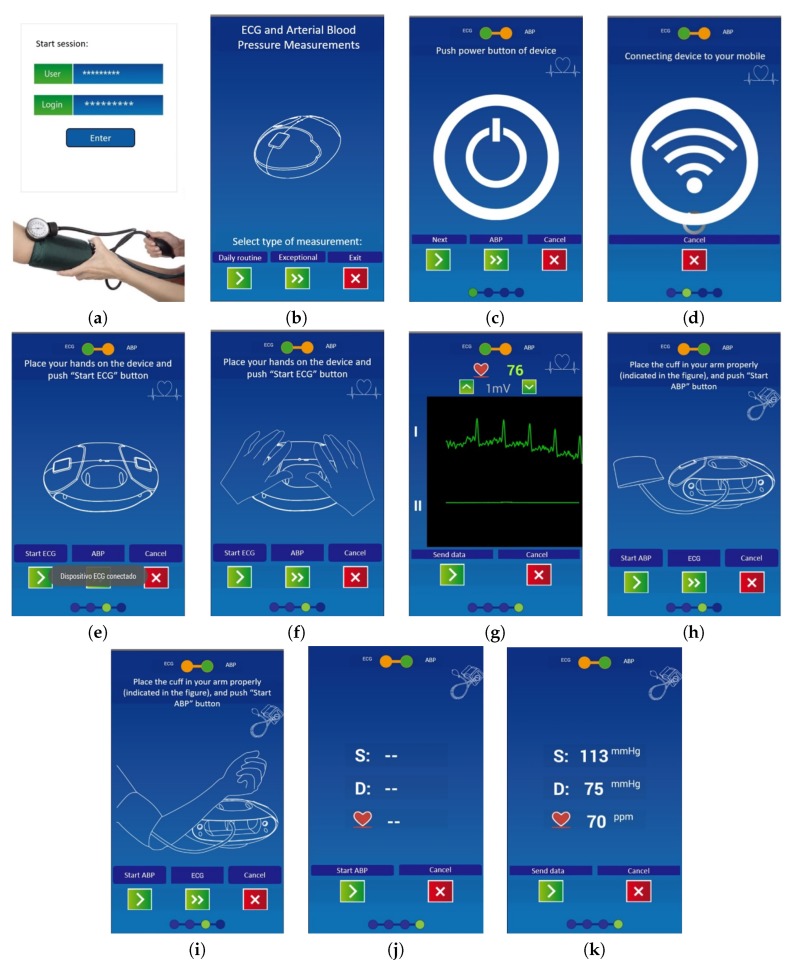
App interface design. (**a**–**d**) Login and initial screenshots. (**e**–**g**) ECG measurement procedure screenshots. (**h**–**k**) ABP measurement procedure screenshots.

The application interface is specifically designed to adapt to the elderly, in a very easy-to-use way. The interface includes big buttons and the essential options, two or three options per screen, with big and high-contrast representative images to clarify some aspects of the device working mode. The app screen upper side contains a brief description of the step of the procedure and it includes a big image at the center describing the next required action. App options and buttons are included on the bottom of the screen and the user navigates through the app by pressing these buttons. Measurement procedures are very easy to follow, the interface includes *next* buttons to continue with the procedure in a very simple mode, as it can be observed in Figure 5.

From a functional perspective, basic processing features were built in the VitalMob App solely to advise the user to perform a new manual registry if it was not possible to obtain a valid processing signal, and to offer basic information such as average heart rate or and blood pressure range for the user convenience.

### 3.3. Remote E-Health Platform

A remote e-health platform has been developed to store and automatically analyze patient health information and to facilitate communication between doctor and patient. For this reason, its generic functionality is ideal for the VitalMob research project, in which the monitoring of the clinical evolution of ECG and ABP of the patient over time is relevant. Some of the advantages of the platform are:

- It is a versatile and configurable platform. It includes questionnaires to collect patient information and support for patient assistance reports.
- It is offered as software as a service, so it is cheaper than other custom tools and has the potential to integrate with any clinical services.
- It allows remote monitoring of patients using different tools such as questionnaires and online consultations.
- The collected data are easily integrated in a database and it is available for download in different formats.
- It includes received signal conditioning filtering, HRV analysis techniques and QRS-complex morphology enhancement, as explained later.

In contrast to the limited processing functionalities incorporated in the home device, the remote e-health platform incorporates all features and advance processing developed by the engineering team, yielding relevant diagnostic information available of-the-shelf for clinician diagnostic support.

In this system, we developed a classical three-stage algorithm to perform the ECG analysis. These three parts are: *Signal adequation*, where different signal processing steps are applied over the signal in order to remove or at least reduce the present noise; *Beat detection*, where a tailored version of the well-known Pan-Tompkins method was developed by us and applied; Finally, *rhythm and morphology analysis*, where the heart rhythm is inspected in order to detect different kinds of arrhythmias and a beat template is created to allows clinician to enhance their morphology visual inspection.

In the signal pre-processing stage, several filters were implemented in order to reduce the present noise. According to Reference [7], we implemented a low-pass filter with a cut-off frequency of 75 Hz in order to preserve the signal morphology. The baseline drift was removed by using a cubic spline interpolation, using as knots the mean value of consecutive signal segments. The powerline interference was reduced by applying a notch filter centered on 50 Hz, which is the powerline frequency in Spain. Finally, the so-called large amplitude noise was found to be present in this type of sensors and it was removed by using a special filter designed for this purpose, which computes the following steps: (i) The signal is divided in blocks of 0.5 s; (ii) The standard deviation in each block is computed; (iii) The mean and standard deviation are computed for every ten previous standard deviation blocks; (iv) Finally, a threshold based on mean plus twice standard deviation is used to remove the noisy blocks.

After reducing the present noise in the ECG, the QRS-complexes are detected in the beat detection stage, according to Reference [13]. The used method is based in feature signal creation, which enhances the morphology of QRS-complexes and to create it, the following filter was applied,

(1) $$H\left(z\right)=\frac{1}{10}\left(-2z^{-2}-z^{-1}+z+2z^{2}\right)$$

After that, a threshold-based on filtered signal mean and standard deviation is applied to select the QRS-complexes location zones. Afterward, each zone is separately processed to find the QRS exact position by searching the maximum amplitude of the segment. Finally, the detected peaks are passed through a simple filter that removes the artifacts, this is, those for which their difference with their neighbor detected peaks are smaller than a specified refractory period.

Finally, in the rhythm and morphology analysis stage, two techniques were developed. After collecting the QRS-complexes exact position, we defined a number of warnings: Rhythm alteration, which represents a RR interval that differs more than a 30% of previous interval; Pauses, which are detected as RR intervals longer than 3 seconds; And AF detection, using the fact that this is an illness characterized by the atria randomly beating and it may causes a worsening in patient quality of live, as well as eventual possible risk of penitential deadly diseases.

The AF detection is performed by using the Lorenz-Poincaré diagram. According to medical knowledge, a randomly distributed Lorenz-Poincaré diagram is a good AF marker [32]. Therefore, we implemented a Lorenz-Poincaré tailored version, so that in this representation the abscissa axis represents the RR interval value, while the ordinate axis shows the derivative of this interval. From an algorithmic stand point, we proceeded as follows: First, the RR are denoised by removing the outlier values, for which we consider a RR interval as outlier when it meets the next conditions,

(2) $$\begin{array}{cc} O_{1}\left[n\right] & =RR\left[n\right]>\left(P_{25}\left(RR\left[n\right]\right)-3\;IQR\left(RR\left[n\right]\right)\right)\wedge RR\left[n\right]>\left(P_{75}\left(RR\left[n\right]\right)+3\;IQR\left(RR\left[n\right]\right)\right)\\ O_{2}\left[n\right] & =dRR\left[n\right]>\left(P_{25}\left(dRR\left[n\right]\right)-3\;IQR\left(dRR\left[n\right]\right)\right)\wedge dRR\left[n\right]>\left(P_{75}\left(dRR\left[n\right]\right)+3\;IQR\left(dRR\left[n\right]\right)\right)\\ O[n] & =O_{1}\left[n\right]\vee O_{2}\left[n\right] \end{array}$$

where $O_{1}\left[n\right]$ represents the *n-th* outlier depending of RR interval, $O_{2}\left[n\right]$ represents the *n-th* outlier depending of RR interval derivative, O[n] represents the *n-th* outlier, RR[n] represents the *n-th* RR interval, $P_{x}\left(y\left[n\right]\right)$ represents the percentile *x* of y[n] for all *n*, IQR(·) represents the interquartil range, dRR[n] represents the RR derivative, and ∧ (∨) represents the AND (OR) logical operator. After RR-interval denoising, the diagram is discretized creating equally sized bins in both axes; Then, the number of points inside each bins is computed; Finally, the disorganization is measured by computing the percentage of full bins, so that the higher values of this coefficient would eventually correspond to higher probability of existence of AF episodes.

In the case of morphology enhancement processing, we applied an algorithm developed by our group, presented in Reference [33]. This stage is performed by a process that creates a low-noise beat template fitting clinical criteria for visual inspection and evaluation. To do so, only properly correlated beats are considered for statistical filtering purposes. The detailed process is describe here after: (i) The beats are split according to the heart rate, selecting the 40% (60%) of total cycle before (after) each detected R wave; (ii) A 10% of total cycle duration window is centered in the R waves, selecting the QRS-segment; (iii) The correlation coefficient is computed for each one of these segments; (iv) Finally, those segments that achieve a coefficient value higher than 99.5% are averaged in order to create the beat template, which is a low-noise waveform where the clinician can evaluate ECG morphology. Note that most of the analysis and detection in the algorithms is made on HRV measurements but the morphology can be subsequently analyzed in these beat templates and in general the complete ECG signal is stored for future medical retrieving when needed.

## 4. Experiments and Results

The performed experiments can be divided in four main parts, namely: *Algorithm testing*, where the algorithms are tested over classical ECG signals in order to validate them; *Electrode testing*, where a new type of ECG electrode is compared with the classical ones; *System comparison*, where the VM1 is compared with the BIOPAC system; And *ambulatory scenario testing*, where the whole system is tested on real patients.

### 4.1. Algorithm Validation

In this set of experiments, we carefully performed the free parameters fine-tuning of previously mentioned algorithms specifically for the device characteristics, in order to meet the clinical requirements given by expert clinicians from University Hospital Virgen de la Arrixaca of Murcia. The ABP technology integrated into the VM1 had been previously validated by RGB manufacturer in precedent works [34,35]. They tested the ABP sensor over 17 adult patients by comparing it with an invasive ABP measurement taken by an intra-arterial catheter. The collected data were analyzed using the Bland-Altman method. The obtained results were 1.6±7mmHg (−3.4±6.3mmHg) mean error and standard deviation of systolic (diastolic) blood pressure. Both results were within the European accuracy requirements for medical devices.

**Figure 6:**
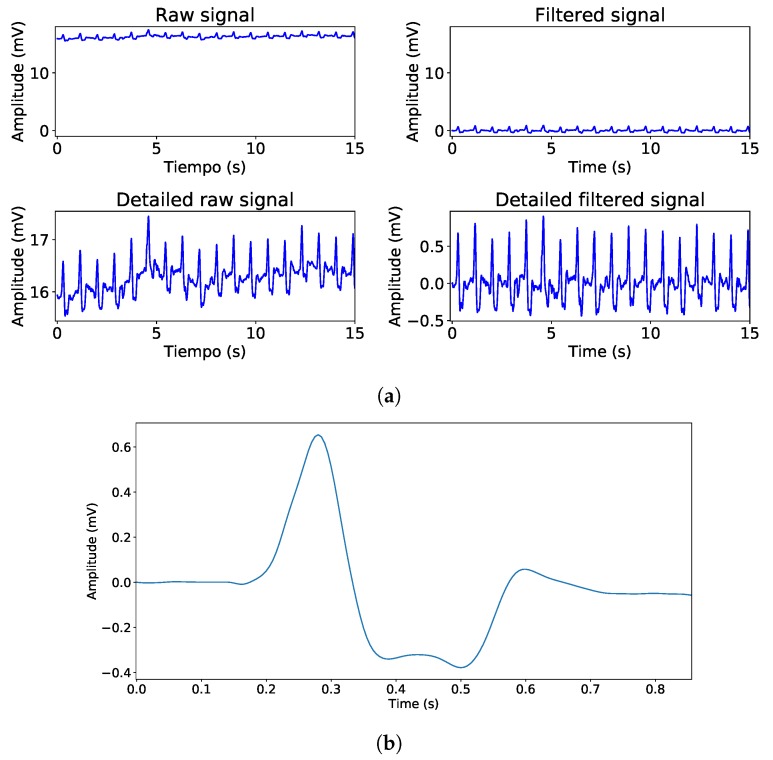
Different implemented filtered effect and beat template. (**a**) Implemented filters effect, Panel 1 shows Raw signal, Panel 2 shows filtered signal, Panel 3 shows detailed raw signal and Panel 4 shows detailed filtered signal. (**b**) Computed beat template for morphology scope tasks.

Figure 6a shows the effects of the different implemented filters on a VM1-recorded signal. As it can be seen in Panel 1 (2), which shows the raw (filtered) signal, the VM1 device induced a DC value around 16 mV that is reduced by the implemented filters. Moreover, Panel 3 (4) that shows the detailed raw (filtered) signal, as the baseline drift observed in Panel 3 is reduced as shown in Panel 4 due to implemented filter, although the low-pass filter performs a moderate noise reduction due to its conservative cut-off frequency value. Figure 6b shows the beat template, which is a low-noise most common morphology presented on the ECG and it can be used to perform several clinical analysis and measurements.

**Figure 7:**
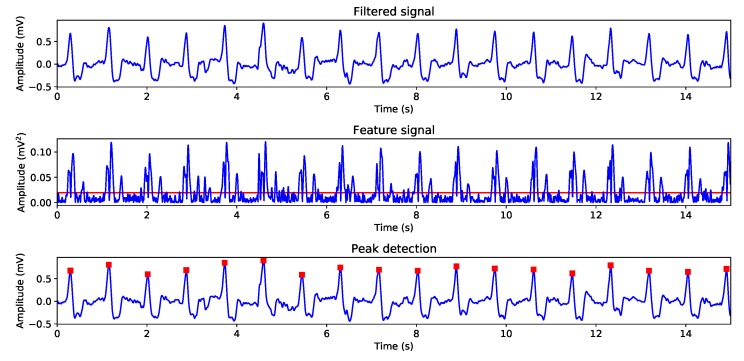
QRS-complexes position detection stages. Panel 1 shows the filtered signal, Panel 2 shows the computed squared features signal, Panel 3 shows the thresholded features signal.

As explained in Section 3, we implemented a tailored version of the Pan-Tompkins QRS-complexes detection algorithm and in this stage we tested different algorithm internal thresholds, selecting the one that achieved better results after careful visual inspection of the detected beats. Figure 7 shows an example of the three main stages of the implemented algorithm, namely: *Filtering*, which is represented in Panel 1 and it is the output of the filter bench previously explained; *Feature signal computation*, which is generated by applying the equation seen in Section 3 over the filtered signal and squaring the results, as it can be seen in Panel 2 where the feature signal appears in blue and the threshold applied appears in red; *Peak detection*, in terms of the described time-amplitude-based thresholding process shown Panel 3, where the amplitude-based threshold selects the regions of QRS-complexes.

**Figure 8:**
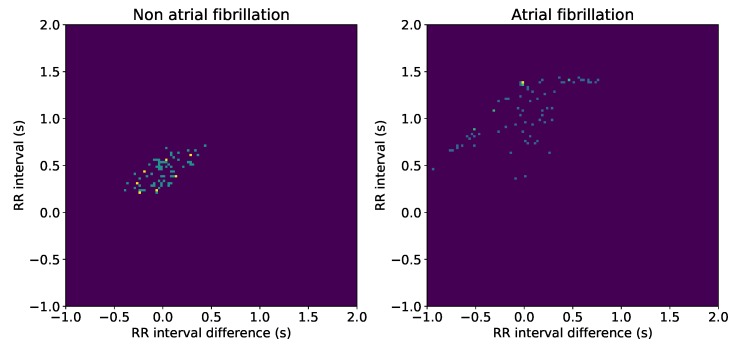
Implemented Lorenz-Poincaré tailored version, where the left panel shows an example of non-AF patient and the right panel shows an example of AF patient. Note the dispersion of the point cloud the AF case compared with the non-AF case.

The AF detection algorithm, which is a customized version of an algorithm previously presented by others [32], was tested over a Holter database that presented different types of arrhythmias. The proposed detector reached 95.3% sensitivity and 88.8% specificity and these values were reached using 75s length segments. Two Lorenz-Pointcaré diagram examples are shown in Figure 8, one for AF and another for non-AF rhythms.

### 4.2. Electrode Experimental Evaluation

On the other hand, the ECG functionality was initially tested with some hardware changes. Although the final VM1 device integrates two metal electrodes for direct contact with the hands, the initial system was tested with capacitive electrodes from Plessey^TM^ for non-direct skin contact measurements. A set of recordings was completed with more than 100 signals with electrodes placed on different body areas, registered at the University Hospital Virgen de la Arrixaca of Murcia under clinical supervision [36]. The computational processes integrated into the remote e-health platform (signal filtering, beat detection algorithm and QRS-wave conditioning) were validated at this initial stage but finally and due to lack of precision and high noise in many of those trials, the electrodes were changed from Plessey^TM^ to metal plate ones.

### 4.3. Initial In-lab Hardware Testing

VitalMob ECG functionality was tested again with more than 20 signals acquired with the final metal plate electrodes. The experimental setup of these tests consisted on acquiring ECG signals from the VM1 and from a BIOPAC system [37] simultaneously.

The BIOPAC system is widely used in the biosignal research field [38,39,40]. The BIOPAC physiological measurement system, so-called BIOPAC MP150, was used to obtain ECG data. Specifically, the ECG amplifier module (ECG100C) was connected to the BIOPAC system to record ECG signals. A three-lead configuration was used for collecting ECG data, so that the white lead was connected to SHIELD and VIN- on the ECG100C module, the red lead was connected to SHIELD and VIN+ and the black lead was connected to GND.

**Figure 9:**
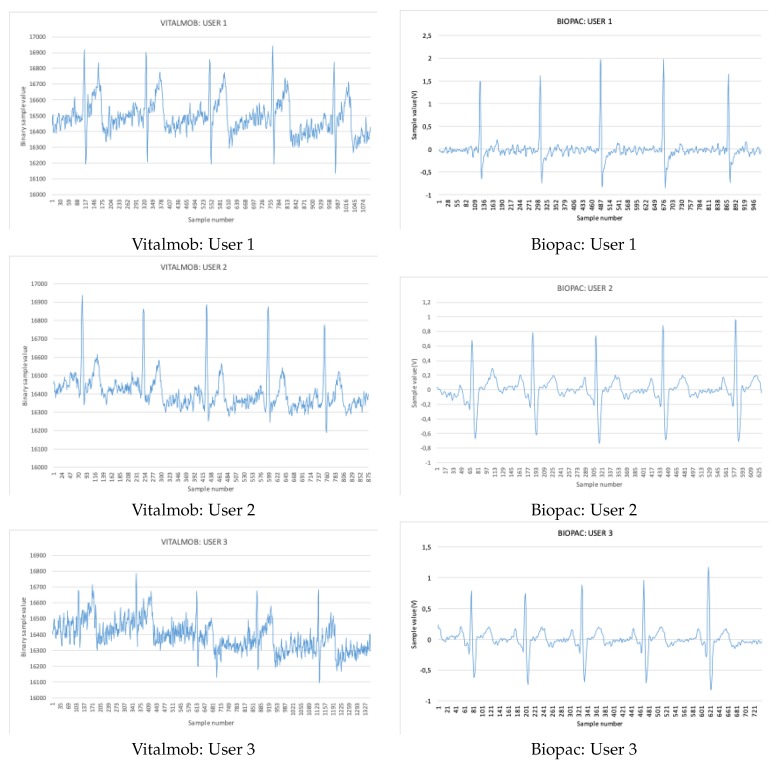
VitalMob and Biopac ECG samples acquired.

During the tests, the users were sitting on a chair. Measurements from VM1 and BIOPAC systems were acquired simultaneously and compared against each other to verify proper VM1 performance. Figure 9 shows three comparison examples among acquired signals from three different users.

### 4.4. Ambulatory Scenario Validation

VitalMob system was tested with 60 patients from the Arrhythmia Unit in the University Hospital Virgen de la Arrixaca of Murcia (HUVA), under clinical supervision. This experiment served to a dual objective: First, it allowed to evaluate the signal quality to perform diagnosis and to validate the algorithms; Second, it also allowed to know how different users, including older adults, interact with the device.

**Figure 10:**
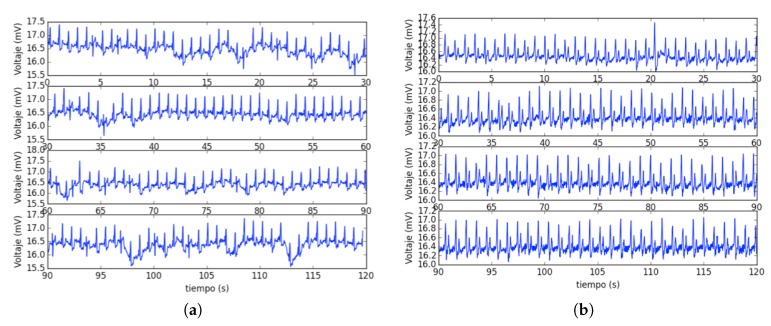
Signal quality depending of how the device is held. (**a**) Device on table and hands on electrodes (higher noise, poorer quality). (**b**) Device held side-by-side by patient with the thumbs on electrodes (lower noise, higher quality).

We noticed that the signal quality is improved if the patient grabs the device from both sides using her/his thumbs to contact the electrodes. In case of putting the hands on the electrodes with the devices on the table, the involuntary tremors of some older patients can induce a transient interference, as shown in Figure 10.

**Figure 11:**
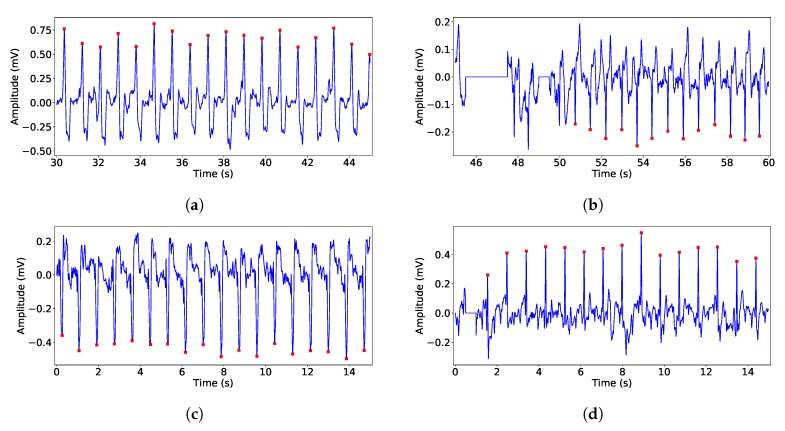

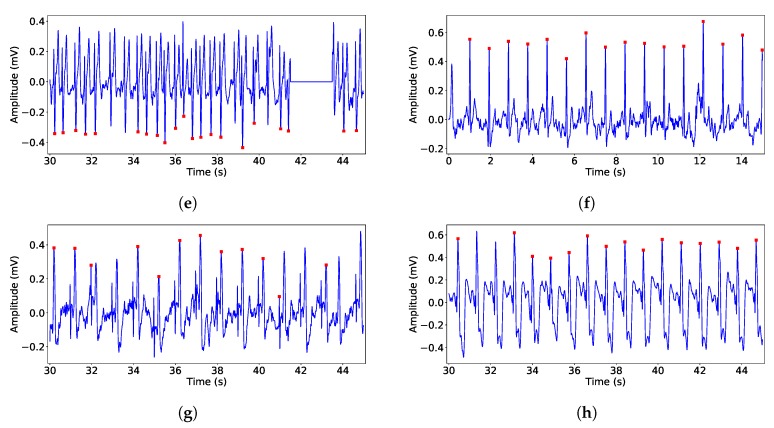
Example of records collected in HUVA: (**a**) Enlarged QRS; (**b**) supraventricular extrasystole; (**c**) Atrial tachycardia; (**d**) Atrial flutter, (**e**) AF, (**f**) Ventricular extrasystole; (**g**) Atrial stimulation pacemaker; (**h**) Ventricular stimulation pacemaker.

After this experiment, we used the second type of grip hereafter. The patients where randomly assembled along several weeks. After recording them, an expert clinician diagnosed each of them, reporting the next types of diseases: 15 records presented normal rhythm patients, 2 records presented atrial arrhythmia, 2 records presented normal rhythm with enlarged QRS, 2 records presented atrial extrasystole, 6 records presented ventricular extrasystole, 4 records presented AF or flutter, 3 patients with implanted ventricular stimulation pacemaker, 1 patient with implanted atrial stimulation pacemaker, 2 patients with non-identifiable rhythm due to EMG noise and 25 healthy control patients. Figure 11 shows an example for several of these diseases, except for the healthy patients, which are not included in the plot due to the low clinical relevance. Note that the worst case is the ventricular extrasystole, due to the sign inversion of QRS-complexes, whereas the AF was detected by our algorithm in the present test subjects.

## 5. Conclusions and Future Work

This paper has presented an overview of a novel telemedicine solution for in-home heart monitoring developed in the research project called VitalMob. Its main objective was to create a very simple, high quality and easy-to-use portable ECG and ABP monitor, with cloud-based automatic analysis within a complete e-health platform, which can be used by people with low technical skills or no medical experience, such as older people and, in general, most heart disease patients. This solution contributes to reduce health-related costs and at the same time it allows self-monitoring, which can be done regularly and as soon as the patient thinks that she/he needs it.

The paper has mainly focused on describing the VM1, a portable device and its companion smartphone app. Much effort has been put in order to make the VM1 highly ergonomic and convenient to use. It is capable of recording high quality ECG signals as well as of measuring the ABP. The VM1 is controlled via a Bluetooth^TM^ LE connection by a smartphone app that guides the user through the multiple steps of the measuring process. Device and app were both jointly designed for maximum ease of use, so that any common older person or cardiac patient could use it without external aid.

The ECG signal can be visualized during its measurement in real time by the user, who can decide whether to upload to the cloud after recording it or to repeat the process if something went wrong. Once in the cloud, data is analyzed by our developed algorithms that look for potential problems and anomalies (e.g, for arrythmias). If anything, unusual is detected, the doctor is warned to inspect the recorded data through any internet-capable device and to visually confirm if it is a real problem and decide how to proceed.

In the most common mode of ECG operation, the user places its hands over the flat electrodes embedded on the top of the VM1 case. This makes the ECG measurement process much easier for non-expert users and although it could compromise some ECG quality in return, it has been proven that it is still sufficient to highlight many common potential problems. If that is the case, then a more complete ECG in the hospital could be prescribed.

Compared to similar solutions, and to our best knowledge, the VitalMob system is the only end-to-end system in the literature that can send ECG and ABP data to the cloud for immediate analysis. Moreover, the VM1 is versatile enough to provide three modes of operation: two embedded electrodes, two wired electrodes and a two-by-two mode uniquely devoted to improve signal quality in special user’s situation. And even more, the existence of up to four simultaneous electrodes open forthcoming potential use cases, as new software releases could define new tailored situation to be identified or isolated in ECG registries in future updates. Under a more functional perspective, this device could be used as a standard domestic tool for frequent ABP and heart rate registry, as well as on-demand event-Holter functioning mode, in both cases for self-monitoring purposes or under clinical supervision. Finally, the VM1 has been validated in two stages under medical supervision and it has also been compared to a very precise reference system for biosignal analysis (Biopac MP150 and ECG100C modules).

The described portable solution is a clear example of an active assisted living (AAL) Sensor-based infrastructure system that contributes to elderly independent and more autonomous living. RGB and CETEM’s R&D teams are already working in the last stages of a new project for enhancing the VM1 so that it can be used to estimate other haemodynamic variables that may shed more light on other cardiovascular pathologies such as heart failure.

The main value proposition of the integrated system solution described is the multifunctional and quadruple result achieved, providing a real-effective solution to an existing need, under a closed perspective and including all the necessary agents, which ended up conducting to an accepted patent. The first aspect, the real need, could be proven by the financial grant provided by the Spanish Ministry referenced below in the acknowledgments section. Regarding the comprehensive and complete vision, it is necessary to point out the proven usability, a sound design, the application and development of the required specific algorithm (signal adaptation, delineation, detection and affection identifications) and the end-to-end clinical validation of the proposed solution-system. Under the usability perspective, the proposed solution has been tested by a commercial company, actively in operation, which has been devoted for years to the development of digital medical service platform and solutions, as well as by a solution-development entity integrating the entire furniture-hub in the region of Murcia. Under the perspective of the sensorization, the team incorporated the cutting edge technology from a company specifically dedicated to sensors and hardware development. Under the perimeter of the signal processing, the proposed solution was developed by the academic-research teams, incorporating the latest generation signal-processing algorithms supported by the experience of decades of research in the electrophysiology field. Finally, the system has been validated, in all its individual elements as well as end-to-end, by the specialized clinical team of the medical services of the reference hospital, ensuring that the results obtained thus meet their requirements.

*Current Limitations and Future Research.* First, it is important to note that the presented device is not a medical device yet, as there are different specifications and certifications from regulatory agencies that need to be passed to be considered as such and the VM1 device is in an initial phase of this path. According to our cardiologists partners, its use as a medical device would be interesting and the effort required for its certification should be considered. On the other hand, signal processing software has been tested not in isolation but rather on an end-to-end system environment and within a clinical embedding. Whereas this gives a convenient scenario for algorithm improvement, nevertheless additional algorithmic advances are to be addressed in the near future. Special attention is being received by the AF detection, for which even competitions and hackatons have been recently proposed to the research community [41]. The existing proposals in the literature include machine learning and advanced deep learning methods, which should be tested in said clinical scenarios to promote their enhancement. On the other hand, the system can perform a prolonged monitoring on a discontinuous way, what can be called extended monitoring (rather than long-term monitoring, which is often understood for continuous monitoring). This kind of systems have demonstrated their usefulness for instance when detecting asymptomatic AF (in the ECG) and in general for ABP monitoring (see e.g., References [42,43] for examples on the clinical usefulness of this extended monitoring). Also, the requirement of real time could be asked to this kind of systems, which can not be guaranteed but rather because of the system implementation and communications. For instance, diagnosis like *AF Suspicion* or *High-pressure Suspicion* are readily achievable with the implemented algorithms but it should be incorporated in the local communication system (for instance to avoid delays due to lack of connectivity) to ensure their immediate diagnosis sending. The delay on the diagnosis establishment will depend in general terms on how much software is integrated in local and in how the system is integrated in the health infrastructure (e.g., technician reviewing the data as soon as they are sent, or once a week, or only in case of alert). The philosophy of the system is not to detect pathology requiring immediate action, for instance, a delay of 24 h is not determining for AF detection or for hypertension management. Finally, if the patient or subject has tremors or is not able to cooperate and to stand still during the measurement, this could be a not-ideal candidate. Nevertheless, most of subjects will be able to use the system with enhanced comfort.

In conclusion, although a large number of ECG-devices are currently being commercially launched to the market, to our best knowledge, there are not so many published papers that integrate a clinically validated system, covering completely the full value chain, which combines ECG recording and ABP measurements, as the one presented in this paper. Above all, it is relevant in this case that the product presented is devoted especially to the elderly for home usage and it is able to generate arrhythmia alarms and to connect with public or private health services.

## 6. Patents

A patent has resulted from the work reported in this manuscript, which is patent number: ES 2 587 789, with patent title: “Portable arterial blood pressure and electrocardiogram measuring device”.
